# Host search behaviors of specialist and generalist root feeding herbivores (*Diabrotica* spp.) on host and non-host plants

**DOI:** 10.1038/s41598-023-44760-w

**Published:** 2023-10-16

**Authors:** Man P. Huynh, Ryan W. Geisert, Dalton C. Ludwick, Adrian J. Pekarcik, Bruce E. Hibbard

**Affiliations:** 1 Division of Plant Science and Technology, University of Missouri, Columbia, MO USA; 2 Department of Plant Protection, Can Tho University, Can Tho, Vietnam; 3 Department of Entomology, Texas A&M, AgriLife Extension Service, Corpus Christi, TX USA; 4 North Central Agricultural Research Laboratory, USDA-Agricultural Research Service, Brookings, SD USA; 5 Plant Genetics Research Unit, USDA-Agricultural Research Service, Columbia, MO USA

**Keywords:** Entomology, Behavioural methods

## Abstract

Western, northern, Mexican, and southern corn rootworms (WCR, NCR, MCR, and SCR) are serious corn pests. We evaluated host search behavior of these pests on six plant species using a video tracking system. After a 5-min exposure to plant roots, behavioral parameters were automatically recorded and used to quantify the search behavior. The search behavior was not observed for sorghum since no neonates survived after contacting sorghum roots. After exposures to corn roots, all neonates exhibited the localized search behaviors (i.e., shortening total distance traveled, lowering movement speed, increasing turn angle, moving farther from origin) which are used to stay in and search within root systems. When larvae contacted roots of wheat, barley, oats, soybean, or controls, they expanded the search area by extending the travel path, increasing velocity, and reducing turn angles and total distance moved. The intensity of the search expansion is highly associated with the host preferences known for the four rootworm species and subspecies. Neonates of each corn rootworm exhibited distinct search behaviors. In fact, NCR larvae had the highest speed, the greatest travel path, and the lowest turn angle, whereas MCR larvae had the highest turn angle and moved faster than WCR and SCR larvae.

## Introduction

Four *Diabrotica* species and subspecies including western corn rootworm (WCR), *D. virgifera virgifera* LeConte, northern corn rootworm (NCR), *D. barberi* Smith & Lawrence, southern corn rootworm (SCR), *D. undecimpunctata howardi* Barber, and Mexican corn rootworm (MCR), *D. virgifera zeae* Krysan & Smith, are economically important insect pests of corn (*Zea mays* L.) in the United States^1,2^, costing over $2 billion annually in economic losses due to management costs and yield reductions^3^. These insect species are native to North America^1^ and are commonly referred to as corn rootworms since their larvae primarily feed on the roots of corn^4^ resulting in most of the damage caused by these pests (e.g., detrimental effects on nutrient and water uptake of corn plants, facilitation of pathogen infection, and reduced plant stability)^5,6^. Several management strategies (e.g., crop rotation, chemical insecticides, transgenic corn) have been developed to manage WCR and NCR; however, both these species have evolved adaptation to nearly all management tactics^7–12^. Additional control tools clearly need to be developed and implemented for sustainable management programs of the corn rootworms.

Western, northern, and Mexican corn rootworm larvae are nearly monophagous on corn roots, while SCR larvae are polyphagous herbivores^13–18^. Female corn rootworm beetles lay eggs in the soil and after overwintering, their newly hatched belowground neonates have limited time and sensory information to search and travel through soil to find suitable host roots^19^. The larval search behaviors typically consist of two phases including ranging, a relatively straight form of locomotion occurring prior to contact with potential host plants, and localized search, a more convoluted track occurring after larvae have gained information on the available resources^20^. These searching mechanisms are crucial for larval survival and fitness^21^. While the straighter and faster ranging behavior increases search efficiency for finding food from far distances, the localized search including shorter paths and slower movements are more efficient to locate food within a food patch^20,22^.

Most of the findings on host search behaviors of corn rootworms are obtained from WCR research, while limited information is available for other corn rootworm species. If they have not found a host, WCR larvae exhibit a ranging behavior in which they can travel in a relatively straight direction for far distances in search of cues from potential host plants^20,23^. Several studies have been demonstrated that WCR larvae utilize volatile and non-volatile chemical cues that are either present on corn roots or are emitted into the surrounding soil. Carbon dioxide released by the plant roots during respiration was found to be highly attractive to WCR larvae^23–25^. Silencing CO_2_ perception in WCR larvae via RNAi-mediated knockdown of a carbon dioxide receptor (*Dvv*Gr2) impaired WCR larval ability to locate corn roots at distances > 9 cm but had no effect on WCR host-locating ability at shorter distances^26^. (*E*)-β-caryophyllene and ethylene are also volatile compounds found to be involved in WCR orientation of 2nd instar larvae^27^, but at least (*E*)-*β*-caryophyllene did not appear to influence behavior of neonate larvae^28^. Additionally, 6-methoxybenzoxazolinone (6-MBOA) and complexes of iron and 2,4-dihydroxy-7-methoxy-1,4-benzoxazin-3-one (DIMBOA) were identified to be involved in host orientation by second instar larvae to locate nutritious roots^29–31^. In addition to the identified attractants, methyl anthranilate was identified in corn roots as a repellent for WCR larvae^32^.

Immediately after contacting plant roots, the WCR response to contact cues dominates over response to volatile cues^20^. WCR larvae can determine its potential as a host or non-host plants based on the detection of various chemosensory cues present in the plant roots with the aid of their maxillary palpi^33,34^. These chemosensory cues can act as either a phagostimulant (e.g., sugars) which encourages feeding or as a feeding deterrent (e.g., phenolic compounds) which would cause the larvae to seek a different feeding site^33^. It has been determined that a feeding stimulant for WCR larvae is a blend of three simple sugars (30:4:4 mg/ml of glucose:fructose:sucrose) plus free fatty acids (2:5 mg/ml oleic acid:linoleic acid)^35^. Later, monogalactosyldiacylglycerol (MGDG) consisting of a glycerol backbone with a single galactose molecule and two fatty acids was discovered as a host recognition cue of WCR larvae^36^.

The host-search behavior of WCR larvae switches from ranging to localized search behavior as soon as the larvae contact the roots of a host plant^20,37^. After contacting host plants (corn and a few grasses), WCR larvae exhibited an increase in turning rate and a decrease in locomotory rate which can facilitate the larvae’s probability of contacting the root system. WCR larvae also exhibited significant host preference response to corn hybrids expressing at least one of the insecticidal toxins from *Bacillus thuringiensis* (Bt) Berliner or a double strain (ds)RNA targeting WCR compared to roots of a non-host plant (oats, *Avena sativa* L.)^38^. Nonetheless, the searching behavior of NCR, MCR, and SCR have never been explored to our knowledge. The goals of the present study were to determine and compare the localized search behavior of four corn rootworm species and subspecies (WCR, NCR, SCR, and MCR) on six plant species including corn, wheat (*Triticum aestivum* L.), barley (*Hordeum vulgare* L.), soybean (*Glycine max* L.), oats, and sorghum (*Sorghum vulgare* Pers.). Exploring basic biology such as the localized search behavior of these rootworm species provides a better understanding of fundamental biology, which may facilitate the development of pest management.

## Results

**Figure 1:**
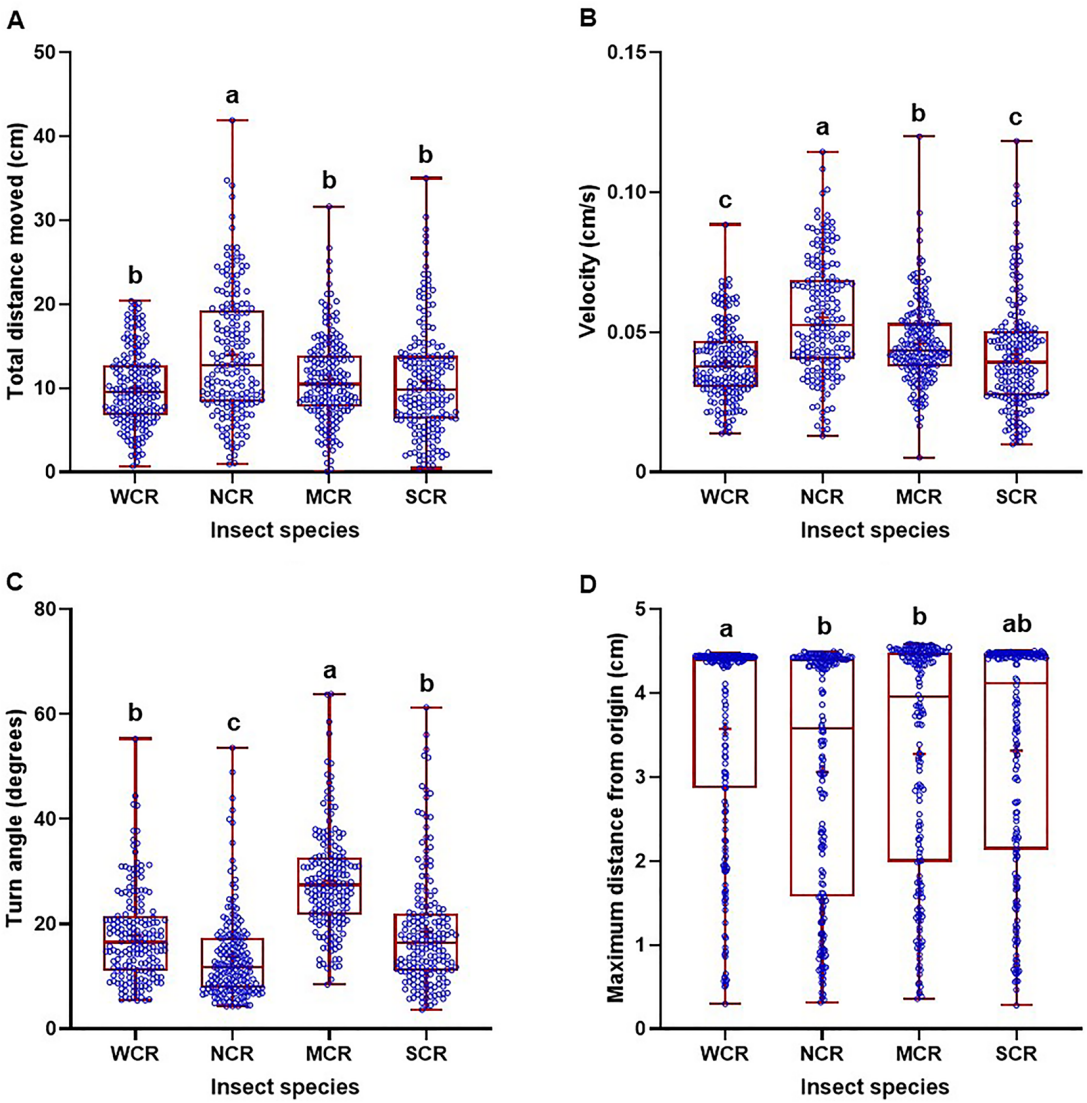
Total distance moved (**A**), velocity (**B**), turn angle (**C**), and maximum distance from origin (**D**) of four larval *Diabrotica* species and subspecies (*WCR* western corn rootworm, *NCR* northern corn rootworm, *MCR* Mexican corn rootworm, *SCR* southern corn rootworm) in 5 min after exposure to different seedlings from five plant species and a blank control. Box plots with median (red line), the 25th and 75th percentiles (bottom and top of box, respectively), the 5th and 95th percentiles (whiskers), and means (+) are shown. Open dots are data points. Boxes with different letters are significantly different (*P* < 0.05). Untransformed data were presented, while analysis was performed with square root transformed data.

**Figure 2:**
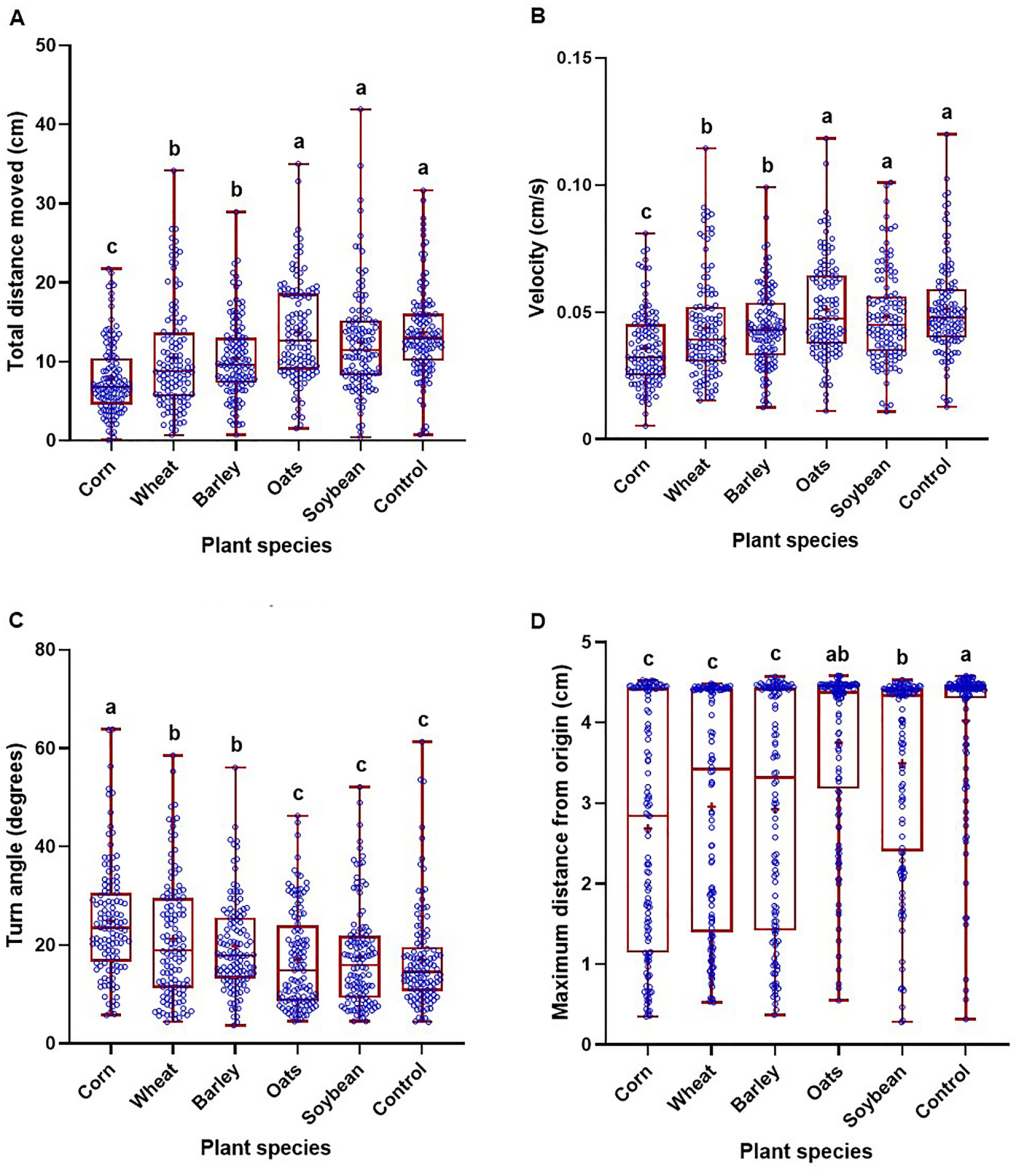
Total distance moved (**A**), velocity (**B**), turn angle (**C**), and maximum distance from origin (**D**) of four larval *Diabrotica* species and subspecies in 5 min after exposure to different seedlings from five plant species and a blank control. Box plots with median (red line), the 25th and 75th percentiles (bottom and top of box, respectively), the 5th and 95th percentiles (whiskers), and means (+) are shown. Open dots are data points. Boxes with different letters are significantly different (*P* < 0.05). Untransformed data were presented, while analysis was performed with square root transformed data.

All corn rootworm neonates exposed to sorghum died within the 5-min exposure period. As such, there were no behavioral data of corn rootworm larvae observed related to sorghum. The search behavior of four corn rootworm species (WCR, NCR, MCR, and SCR) were determined on five different plant species (corn, wheat, barley, soybean, and oats). Significant differences in the search behaviors were found among insect species, plant species and their interactions (Table 1). Generally, WCR and SCR neonates had somewhat similar search behaviors when data from the plant species and control were combined, which were significantly different than NCR and MCR neonates (Fig. 1). WCR and SCR neonates had significantly shorter paths, moved slower, turned more, and traveled farther from the distance from the origin than NCR neonates (Fig. 1). NCR larvae had the longest path and the highest speed, and the lowest turn angle, whereas MCR larvae had the highest turn angle and moved faster than WCR and SCR larvae (Fig. 1). With respect to the plant species with all rootworm species combined, corn had the shortest distance and the lowest velocity and turn angle of rootworm neonates, followed by wheat and barley, and then oats, soybean, and control (Fig. 2). Rootworm neonates that were exposed to oats and soybean moved significantly farther from the origin than those exposed to corn, wheat, and barley (Fig. 2).

**Table 1:** Effect of treatments on behavioral parameters measured of the movement of four corn rootworm species and subspecies during bioassays.

Analysis	Effect	*df*	F value	*P* value
Total distance moved	Insect	3667	15.31	< 0.0001
	Plant	5667	23.62	< 0.0001
	Insect × Plant	15,667	5.99	< 0.0001
Velocity	Insect	3667	35.13	< 0.0001
	Plant	5667	18.29	< 0.0001
	Insect × Plant	15,667	5.81	< 0.0001
Turn angle	Insect	3667	95.79	< 0.0001
	Plant	5667	16.01	< 0.0001
	Insect × Plant	15,667	4.03	< 0.0001
Maximum distance from origin	Insect	3667	4.97	0.0020
	Plant	5667	20.76	< 0.0001
	Insect × Plant	15,667	4.28	< 0.0001

### Western corn rootworm

**Figure 3:**
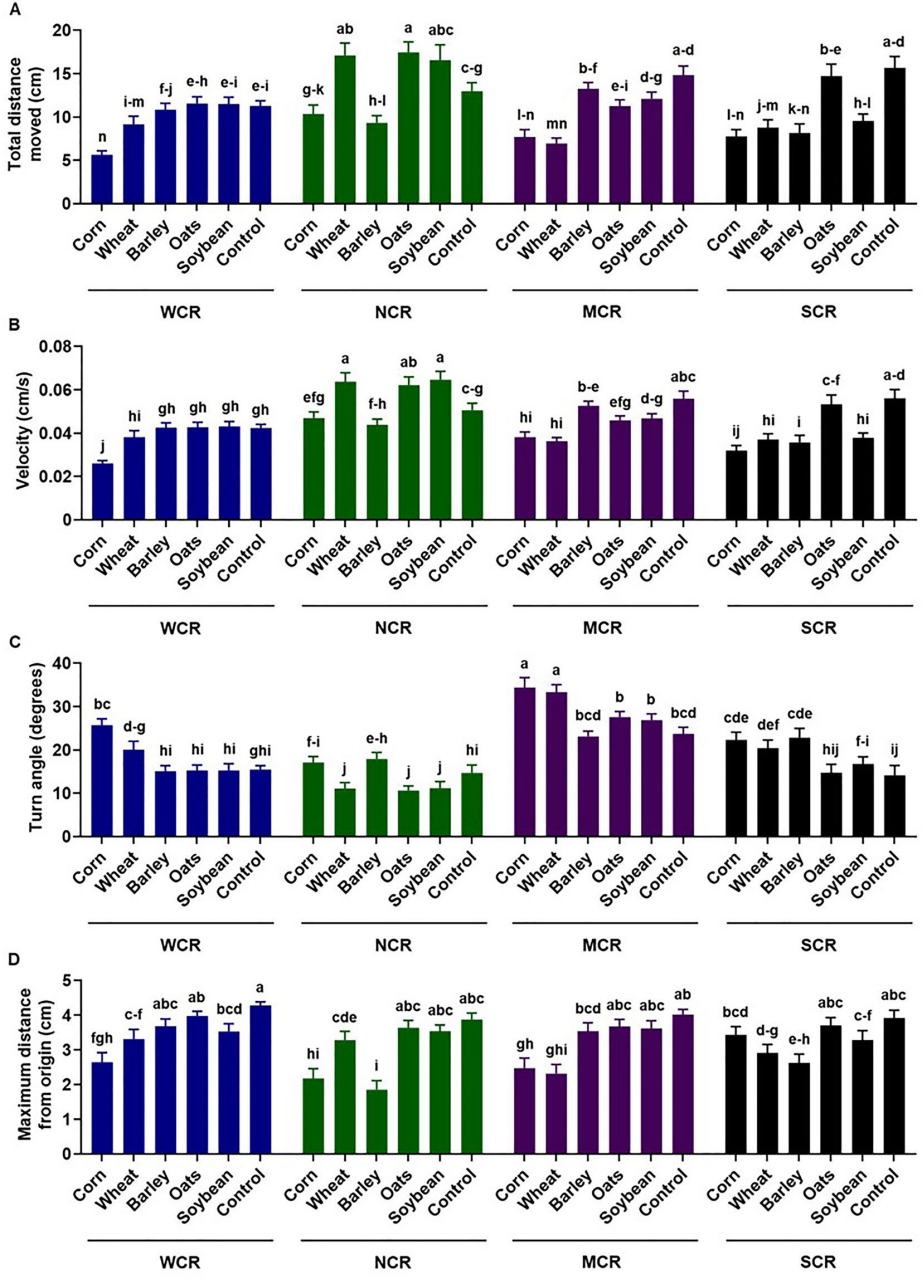
Total distance moved (**A**), velocity (**B**), turn angle (**C**), and maximum distance from origin (**D**) of four larval *Diabrotica* species and subspecies (*WCR* western corn rootworm, *NCR* northern corn rootworm, *MCR* Mexican corn rootworm, *SCR* southern corn rootworm) in 5 min after exposure to different seedlings from five plant species and a blank control. Bars with different letters are significantly different (*P* < 0.05). Mean ± SEM.

WCR neonates had the shortest total distance moved on corn (5.62 cm), followed by wheat (9.16 cm) and barley (10.83 cm), and then oats (11.53 cm), soybean (11.48 cm), and control (11.26 cm) (Fig. 3). The velocity of WCR neonates was significantly lower for those exposed to corn (0.02 cm/s) than those exposed to wheat (0.03 cm/s), barley (0.04 cm/s), oats (0.04 cm/s), soybean (0.04 cm/s), and control (0.04 cm/s) all of which were not significantly different from one another. The turn angle was the greatest for neonates exposed to corn (29.7°), followed by wheat (20.07°), control (15.45°), soybean (15.24°), oats (15.25°), and barley (15.10°). The maximum distance traveled from the origin was highest for neonates exposed to control (4.28 cm) and oats (3.97 cm), while that for neonates exposed to corn (2.64 cm) was significantly lower than all other exposures, except for wheat (3.31 cm) (Fig. 3).

### Northern corn rootworm

NCR neonates had shorter total distance moved on barley (9.33 cm), corn (10.36 cm), and control (12.97 cm) than soybean (16.56 cm), oats (17.44 cm), and wheat (17.09 cm) (Fig. 3). The velocity of NCR neonates was lowest for those exposed to barley (0.043 cm/s), corn (0.046 cm/s), and the control (0.05 cm/s). These were significantly lower than larvae exposed to soybean (0.06 cm/s), wheat (0.06 cm/s), and oats (0.06). NCR neonates had significantly higher turned angle on barley (17.89°) and corn (17.07°) than other plant species tested. The maximum distance traveled was significantly lower for neonates exposed to corn (2.17 cm) and barley (1.85 cm) than those exposed to wheat (3.28 cm), oats (3.63 cm), soybean (3.64 cm), and control (3.87 cm) (Fig. 3).

### Mexican corn rootworm

The total distance traveled was shortest for MCR neonates exposed to wheat (6.96 cm) and corn (7.71 cm), followed by oats (12.50 cm), soybean (13.44 cm), barley (14.73 cm), and control (16.48 cm) (Fig. 3). MCR neonates had significantly lower velocity on wheat (0.03 cm/s) and corn (0.03 cm/s) than oats (0.04 cm/s), soybean (0.04 cm/s), barley (0.05 cm/s), and control (0.05 cm/s). Neonate larvae exposed to corn (34.38°) and wheat (33.35°) had significantly higher turn angle than those exposed to oats (27.58°), soybean (26.87°), barley (23.08°) and the control (23.07°). The maximum distance traveled was lowest for larvae exposed to wheat (2.31 cm) and corn (2.47 cm), followed by barley (3.54 cm), oats (3.67 cm), soybean (3.62 cm) and control (4.02 cm) (Fig. 3).

### Southern corn rootworm

SCR neonates had significantly lower total distance moved on corn (7.75 cm), barley (8.14 cm), wheat (8.77 cm), and soybean (9.56 cm) than oats (14.71 cm) and control (15.67 cm) (Fig. 3). Significantly lower velocity was observed in neonates exposed to corn (0.03 cm/s), wheat (0.03 cm/s), barley (0.03 cm/s), and soybean (0.03 cm/s) than those exposed to oats (0.05 cm/s) and control (0.05 cm/s). The turn angle was greater for neonates exposed to barley (22.79°), corn (22.30°), and wheat (20.39°) than those exposed to soybean (16.76°), oats (14.74°), and control (14.15°). The maximum distance traveled by SCR larvae were greatest on control (3.92 cm), followed by oats (3.70 cm), corn (3.43 cm), soybean (3.28 cm), wheat (2.91 cm), and barley (2.62 cm) (Fig. 3).

## Discussion

In the present study, we documented the search behaviors of four corn rootworm species and subspecies (WCR, NCR, MCR, and SCR) on five plant species including their natural host plant (corn). After contacting roots of corn, corn rootworm larvae exhibited localized search behaviors (i.e., shortening total distance traveled, lowering movement speed, increasing turn angle, moving farther from distance from origin) which are used to stay in and search within the root system. When specialist larvae contacted the roots of other plant species (i.e., wheat, barley, oats, soybean) or controls, they tended to expand their search area via extending the travel path, increasing velocity, and reducing turn angles and the distance moved. Interestingly, neonates of each corn rootworm showed distinct search behaviors. In fact, NCR larvae had the highest speed, the greatest travel path, and the lowest turn angle, whereas MCR larvae had the highest turn angle and moved faster than WCR and SCR larvae. These inherent search patterns may arise from the differences in genotype of these species. A similar pattern of the localized search was previously observed for WCR larvae^20,37,38^. The authors found WCR larvae established the localized search after contact with corn, but not after contact with non-host plants (e.g., oats, soybean).

Our results revealed that the intensity of the search expansion is highly associated with the host preferences of all four corn rootworm species and subspecies. WCR, NCR, and MCR larvae are root-feeding specialists that feed almost exclusively on plant species in the Poaceae family (e.g., corn, wheat, barley)^13–17^, while SCR larvae are root-feeding generalists that can feed on many plant species including those in the Poaceae and Fabaceae (e.g., soybean) families^16,39^. WCR larvae had the most restricted and convoluted search pattern on corn, followed by wheat, barley, soybean, and oats, while no significant differences on the search patterns of SCR were found on corn, wheat, barley, and soybean. NCR and SCR larvae exhibited similar search patterns on soybean and oats. NCR larvae had a more restricted search pattern on corn and barley than wheat, whereas there were no significant differences in the search patterns on corn and wheat for MCR larvae. These behavioral results indicated that corn is the best host plant for WCR, NCR, MCR, and SCR larvae, followed by wheat and barley. In contrast, WCR, NCR, and MCR did not prefer soybean and oats. This agrees with what is known of the host preferences for these insect pests.

After contact with plant roots, corn rootworm larvae utilize contact cues to locate host plants and acquire nutrients, whereas volatile cues are limited to the time of perception^20^. Plants contain nutrients (e.g., amino acids, carbohydrates) and secondary metabolites (e.g., benzoxazinoids) required for herbivore development^30,40,41^. These nutritional factors can serve as contact cues and vary among different plant species, within a plant species, and across genotypes^42^. It is likely that WCR has adopted to corn^43^, but even more so since corn has been widely planted. Indeed, LeConte^44^ first collected WCR in western Kansas. According to Goodman and Galinat^45^, "At the time of European colonization of the New World, maize was being grown from southern Canada to central Chile, although little was grown in the grassy plains or savannas of the central U.S. and northern Argentina". The distribution map of Weatherwax^46^ indicates that indeed maize was not grown in western Kansas in the 1860s. Although something other than corn was producing WCR beetles in the 1800s, it is noteworthy that corn is currently the only known host plant of WCR in nature^43^. Our previous studies demonstrated that WCR beetles can be produced experimentally on a number of non-corn hosts^13,47–49^, though no natural production of WCR has been documented without corn^43^.

Corn roots contain not only feeding stimulants^35,36^ but also likely provide the best-fit nutrients for corn rootworm development. When roots of both species are present, later instar WCR larvae choose to eat corn roots even those expressing insecticidal toxins (Bt corn) rather than eating one of the better alternate hosts^49^. The differences in the search patterns of the specialist root-feeding larvae, that share intimate coevolution adaptation with these host plants^50^, may reveal that neonates of these species acknowledge their best-fit nutrients. Our results indicated that the larvae continued to expand their search intensity to find the better host plants if necessary. Corn rootworm larvae did not prefer roots of oats and soybean. Oats, which contain a feeding deterrent^34^, and soybean are not larval host plants of WCR, NCR, and MCR^16,39^. As a result, the larvae opened their search pattern more intensively compared to other plant species.

The balance between nutrients and secondary plant chemistry often designed to be toxic or repellent to herbivores^51^ is one that has been ongoing since the earliest of times^52^. For the generalist (SCR), there was no difference between corn, wheat, barley, and soybean for several of the parameters measured (Fig. 3). For the specialists, corn and at most one other grass were grouped as having data suggesting preferred status. For all insect species, oats were not preferred. If factors from oats making it a non-host could be identified and placed into the corn genome, not only corn would be a nonhost for WCR, but also for other insect pests evaluated.

## Materials and methods

### Experimental approach

The localized search behavior of four corn rootworm species and subspecies was evaluated on six plant species including corn, wheat, barley, soybean, oats, and sorghum. These plant species were selected based on our previous studies on host preferences of WCR larvae^13,15^. The roots included those of WCR natural host plant (corn), two inferior host plants (wheat and barley), and three non-host plants (soybean, oats, and sorghum). The four insect species and subspecies were exposed as neonate larvae to seedlings from each of six plant species and a blank control for 5 min as described below. After the 5-min exposure, behavioral parameters (i.e., total distance moved, velocity, turn angle, and the farthest distance that larvae traveled) were automatically detected and recorded for 5 min using a video tracking system, EthoVision system 3.1 (Noldus Information Technology, Wageningen, the Netherlands).

### Insects

Eggs from a non-diapausing WCR strain were obtained from the USDA-ARS Plant Genetics Research Unit in Columbia, MO. This strain was derived from a non-diapausing strain originally purchased from Crop Characteristics (Farmington, MN) as described previously^53^. Eggs from a non-diapausing NCR strain were obtained from the USDA-ARS North Central Agricultural Research Laboratory in Brookings, SD. The NCR strain had been selected for a non-diapausing trait from a diapausing strain through selective breeding^54^. Eggs of SCR were purchased from Crop Characteristics. This strain was derived from non-diapausing field strains and was maintained on corn for > 100 generations. The eggs of these species were obtained in Petri dishes containing eggs and soil and then were incubated at 25 °C in complete darkness in an incubator (Percival, Perry, IA). After approximately 5% of eggs hatched, the eggs were washed from soil with tap water and were pipetted onto a coffee filter paper (Pure Brew, Rockline Industries, Sheboygan, WI) placed inside a container with a lid (11.7 × 7.62 × 9.6-cm, LG8RB-0090 & DM16R-0090, Solo Cup Company, Lake Forest, IL) as described previously^55,56^. The eggs were then incubated at 25 °C in darkness and larvae that hatched within 24 h were used for insect behavioral assays.

Adults of MCR beetles were collected from corn fields located in Williamson County, Texas. The field had a history of rootworm damage with continuous corn planting for the past 10 years with the surrounding area being primarily continuous corn. The MCR beetles were reared in the USDA-ARS Plant Genetics Research Unit. The beetles were provided artificial diet^57^, water, and Petri dishes filled with soil as an ovipositional medium to collect eggs^58^. The Petri dishes containing eggs and soil were watered to get 15% moisture (w/v). The egg dishes were then sealed using Parafilm and maintained at room temperature for 9 months. After 9 months, the egg dishes were monitored regularly to collect newly emerged neonates (< 24 h old) that were found moving on the soil plates for use in the behavioral assays.

### Plant materials

Corn (a non-transgenic line) and sorghum seeds were purchased from Albert Lea Seed (Albert Lea, MN) and Green Cover Seed (Bladen, NE), respectively. Seeds of wheat, barley, soybean, and oats were purchased from Johnny’s Selected Seeds (Winslow, ME). Seeds were grown on germination paper covered in a plastic sleeve. Water was added to each plastic pouch to fully soak the germination paper. The sleeves were then held upright with the aid of a stand. Roots were allowed to grow down into the filter paper to provide root material for the experiments. All plant seedlings used in the assays were 3–5 days old with approximately 1.5–2 in. in length.

### Insect behavioral assays

Behavioral assays were performed as described previously^20,37,38^ with modifications. The rootworm search behaviors were recorded and analyzed using EthoVision system 3.1. This system utilized a Monochrome GigE camera mounted 52 cm above an observation arena to autodetect and record movement features of insects. The observation arena was designed as a 9-cm diameter Petri plate lid lined with filter paper. In the Ethovision program, DanioVision settings were selected for the observation.

Fresh germinated seedlings were placed in a Petri dish lined with filter paper. A single neonate larva was then placed directly onto the roots using a fine paintbrush. The larvae were allowed to be exposed to the root material for 5 min. For the control, larvae were placed in a Petri dish with a moist filter paper using purified water and without plant roots. After the 5-min exposure, the larvae were then immediately transferred to the center of the observation arena in the EthoVision system using the fine paintbrush and their movements were automatically recorded for 5 min by the EthoVision system. After the observations, the larvae and root material were discarded, whereas the paintbrush was cleaned with 70% ethanol. Newly hatched and unexposed neonates (< 24 h old) and fresh root material were used for each observation. Each rootworm species was exposed to the roots of six different plant species. The observation was replicated 30 times for each plant species.

Insect behavioral parameters measured included velocity (cm/s), turn angle (degrees, the change in movement direction), maximum distance from the starting point (cm, the farthest distance from the origin that larvae moved), and total distance traveled (cm) during the bioassays using the EthoVision system. In the DanioVision setting, the absolute setting was selected to optimize data collection for small animals (e.g., insects). In the event of larvae moving outside the barriers of the arena after over 90% of time exposure, the total distance was calculated by multiplying the recorded data with the remaining recording time left.

### Data analysis

The experiment was designed as a 2-factor factorial design (insect species × plant species). The behavioral data (total distance, velocity, turn angle, and maximum distance from origin) were analyzed with analysis of variance (ANOVA) in generalized linear mixed models (PROC GLIMMIX) in SAS 9.4 (SAS Institute, Cary, NC). Insect and plant species were the fixed effects and replication was the random variable. Differences between treatments were determined using Fisher’s least significant difference (LSD) at *P* < 0.05. The data were square root transformed prior to the analysis to meet assumption of normality and homoscedasticity, whereas untransformed data were presented as Mean ± SEM.

### Ethics approval and consent to participate

The experimental research and collection of plant and insect materials of this study comply with the relevant institutional, national, and international guidelines and legislation.

## Data Availability

All pertinent data are found in the figures and tables. Requests for data and additional information should be submitted to the corresponding authors.
